# Implementation of evidence-based practice guidelines to prevent urinary retention in hospitals: A process evaluation in orthopaedic care

**DOI:** 10.1371/journal.pone.0338755

**Published:** 2026-09-23

**Authors:** Maria Hälleberg-Nyman, Erika Fjordkvist, Eva Joelsson-Alm, Madeleine Winberg, Ami Hommel, Joan Ostaszkiewicz, Lars Wallin, Ann Catrine Eldh

**Affiliations:** 1 Department of Orthopaedics and School of Health Sciences, Faculty of Medicine and Health, Örebro University, Örebro, Sweden; 2 University Health Care Research Centre, Faculty of Medicine and Health, Örebro University, Örebro, Sweden; 3 Department of Clinical Science and Education and Department of Perioperative Care and Intensive Care, Södersjukhuset, Karolinska Institutet, Stockholm, Sweden; 4 Department of Health, Medicine and Caring Sciences, Linköping University, Linköping, Sweden; 5 Department of Care Science, Malmö University, Malmö, Sweden; 6 The Clinical Gerontology Division, National Ageing Research Institute, Parkville, Victoria, Australia; 7 The Faculty of Medicine, Dentistry and Health Sciences, The University of Melbourne, Parkville, Victoria, Australia; 8 Health and Innovation Transformation Centre, Federation University, Ballarat, Victoria, Australia; 9 Department of Health and Welfare, Dalarna University, Falun, Sweden; 10 Department of Public Health and Caring Sciences, Uppsala University, Uppsala, Sweden; Phramongkutklao College of Medicine, THAILAND

## Abstract

**Background and objectives:**

National clinical practice guidelines promote evidence-based practice, though are hardly implemented without local facilitation. While further research is needed as for what facilitation strategies work, in what context, and with what outcomes, the Onset PrevenTIon of urinary retention in Orthopaedic Nursing and rehabilitation (OPTION) trialled a tailored implementation intervention for evidence-based clinical practice guidelines in orthopaedic care. Informed by the principles of realism, this paper describes the evaluation of the context, mechanisms, and outcomes triad of locally assembled teams of nursing and rehabilitation staff and their managers offered monthly guideline implementation support for one year.

**Methods:**

A process evaluation incorporating group interviews with designated teams across seven intervention sites, at the end of their programme and 12 months later. Individual facilitator logs, photos and educational material from the intervention, and external facilitators’ notes were added. The data was thematically analysed, describing and contrasting the initial hypotheses with a refined understanding of the theoretical and practical features of the process and outcomes.

**Results:**

With increased knowledge of the content and purpose of the guidelines and further recognition of their local context and implementation essentials, the intervention facilitated the local, designated teams to practise concurrent quality improvement procedures and skills. Having both staff and managers on the teams authorised modifications of their local context in favour of guideline implementation. The one-year support programme enabled primary implementation steps, though a comprehension of what could work and why required that the teams saw the intervention through.

**Conclusion:**

Learning about the implementation of a clinical practice guideline is a local effort. This requires dedicated teams with sustained roles, resources, and mandate, with external implementation and clinical support. Nevertheless, this is a slow process that requires repeated or prolonged follow-up to identify long-term implementation intervention outcomes.

**Trial Registration:**

US National Institutes of Health Clinical Trials Registry: NCT04700969. Registered 8 January 2021. https://clinicaltrials.gov/study/NCT04700969.

## Introduction

Across the globe, clinical practice is expected to change with the influx of new evidence into the healthcare sector. Every so often, the new evidence is assembled into updated national clinical practice guidelines (CPGs), which are disseminated to healthcare institutions and professionals who are expected to recognise them, and implement them. However, simply having access to the guidelines do not ensure that they will be implemented. Rather, tailored facilitation is essential [1]. What makes CPG implementation strategies effective may vary with the content and format of the CPG, as well as the context in which they are to be used [2]. Evaluation of CPG implementation calls for recognition of the potential complexity in both processes and outcomes [3].

Healthcare contexts with advanced and highly structured care processes pose certain challenges for implementing CPGs [4]. For example, swift surgical care limits the time patients spend in hospital and thus decreases certain risks while posing others, reinforcing the need for multiprofessional efforts in patient care [5]. Even with effective routines, certain aspects require attention in order to provide both evidence-based and person-centred support to patients [6,7]. Standardised surgical procedures and corresponding nursing and rehabilitation actions need to include opportunities for patients to engage in their own health and healthcare [8,9].

In particular, there is a call for further evidence-based nursing and rehabilitation care associated with hip surgery. In orthopaedic care there is a risk of urinary retention (UR) for patients undergoing hip surgery due to pain, restricted physical ability, and anaesthetics [10]. Several studies suggest the incidence of UR in patients undergoing hip surgery (due to hip fracture or arthrosis) vary, from circa 40% [11–14], to up to 84% [12,15]. Failure to identify and act on UR poses a risk for bladder distention, with further short- and long-term conditions varying from urinary incontinence to sepsis and death [16]. To minimise these risks, current evidence is collated in a national CPG for bladder care in Sweden. This CPG has been freely available for the last decade, and is regularly updated. The CPG indicates when and how to assess the risks of UR after orthopaedic surgery, and what actions should be taken in specific circumstances. However, nursing and rehabilitation staff are not yet fully aware of the risk factors [17], and further efforts are required to translate the CPG into practice [18].

To facilitate the adoption of CPGs by staff and organisations [19], literature about knowledge implementation advocates appointing, training, and supporting staff by setting up local teams of internal facilitators (IF) to promote implementation [20]. However, there is a knowledge gap regarding the optimal construct of such IF teams, and the extent and format of training and support they require to translate CPG knowledge into action.

Few studies have addressed CPG implementation in the orthopaedic context. Following a pilot study reinforcing the need to strengthen implementation efforts [17,18], the Onset PrevenTIon of urinary retention in Orthopaedic Nursing and rehabilitation trial (OPTION) was launched in 2021 [21]. It was based on the assumptions that successful implementation of evidence into practice is a function of the quality and type of evidence, the characteristics of the context, and the way in which the evidence is facilitated into practice [22]. The OPTION implementation intervention built on two determinant frameworks: the integrated-Promoting Action on Research Implementation in Health Services (i-PARIHS) [23] and the Ottawa Model of Implementation Leadership (O-MILe) [24]. The latter was added in recognition of the need for local support from managers to initiate, complete, and sustain implementation.

The 12-month OPTION intervention was delivered by external facilitators as an education and support programme for IF teams, incorporating their first-line managers. To understand how this implementation intervention worked, when, where, and why (or why not), the process was captured and evaluated, with reference to the principles of realist evaluation (RE) [25,26]. This included describing the context, the mechanisms, and the outcomes, framed as CMO triads (for Context – Mechanisms – Outcomes). The CMOs of OPTION were fostered by theoretical assumptions of the i-PARIHS framework [23], including successful implementation of evidence into practice being dependent on the alignment of the evidence with the context through facilitation. In addition, the O-MILe also informed the theoretical assumptions, proposing that implementation is dependent on facilitative leadership, including relations-, change-, and task-oriented behaviours [24]. The core elements of the i-PARIHS framework primarily informed four CMOs and O-Mile added elements to the third CMO, and exclusively constituted a fifth, as in Table 1. Consequently, the i-PARIHS constituted the overarching implementation and facilitation framework, and O-MILe added a specific leadership perspective.

**Table 1:** Overview of the initial hypotheses, with corresponding theoretical assumptions.

Initial hypotheses	Based on core framework principle(s)	Translated into OPTION intervention principle(s)
**I**. Internal facilitator (IF) teams with committed staff with an interest in quality and safety of care and/or the subject of the guidelines (C), recognising external support for a year (M), make use of key steps in a change process (O).	**Adoption of evidence is a non-linear process, activated by facilitators and taking place over time, while incorporating local priorities and practice** [23].	A programme building on common improvement know-how whilst adding implementation of evidence-based guidelines takes at least 1 year for IF teams to recognise and advance the implementation process.
**II**. Known, contemporary, national clinical practice guidelines (C), being recognised for their importance for good and safe practice by the IF teams along with the principles for knowledge implementation (M), become a priority for the IF teams while considering the challenges of, and the necessary elements for, guideline implementation (O).	**Facilitation by enabling others to act is an ideal way in which to embrace processes that recognise and adapt to the dynamic and situation-specific nature of implementation** [23].	The combination of a review of current guidelines – emphasising the importance of knowledge for good and safe practice – and knowledge of implementation processes and their context conditions provides the IF teams with an understanding of the challenges of improvement work and possible success factors.
**III**. First-line managers on the IF teams (C) recognising their role in knowledge implementation (M) enables modification of the necessary local context factors (O).	**Implementation and its facilitation is a group effort, influenced by social networks and leadership enacted in response to people-, task-, and change-oriented conditions** [23,24].	Local teams made up of committed staff – with an interest in quality and safety of care and/or the subject area addressed by the project’s guidelines – and their first-line managers create a mandate to identify and modify or capitalise on the necessary local contextual factors.
**IV**. IF teams (C) interacting with equivalent teams in external, supported events (M) understand local barriers and enablers and address them in guideline implementation (O).	**Implementation and its facilitation is a group effort, influenced by social networks** [23].	Joint IF team meetings between intervention units help them identify their local barriers and enabling factors and address these when implementing guidelines.
**V**. First-line managers on the IF teams in specific forums for sharing knowledge and experiences (C) recognising implementation leadership (M) understand their own role and tasks in implementation in relation to the organisation, the local IF teams, and staff (O).	**Front-line leaders require knowledge of effective leadership practices, skills to prioritize change, and to engage staff for successful implementation of site-specific evidence** [24].	Common forums with knowledge on implementation leadership and exchange of experiences for first-line managers will help them understand their role and task in improvement work in relation to the organisation and the local IF team and other staff [24].

Accordingly, this paper presents the evaluation of the OPTION trial, describing the process and ‘why’ of programme effects [25,26]. The aim was to evaluate what worked, for whom, and in what context, why (or why not), and with what outcomes, in terms of facilitating implementation of evidence-based practice guidelines for bladder care in orthopaedic nursing and rehabilitation.

## Materials and methods

### Design

The process evaluation [27] was inspired by a realist approach as suggested by Pawson, recognising the interplay of context, mechanisms, and outcomes [25]. Several data sources were triangulated to highlight various aspects of the implementation intervention and to support the emerging understanding of the findings. This included data collected at different time points, from diverse situations, representing an inside and outside perspective, and in various formats [28]. In addition, we operated investigator triangulation, with three of the authors engaged in the implementation intervention and two separate authors collecting data, blinded from the intervention content and structure, for neutrality.

The study was registered US National Institutes of Health Clinical Trials Registry (NCT04700969) and reported per the Standards for Reporting Implementation Studies (StaRI) Statement [29] and RAMESES II guidelines for reporting REs [30].

Ethics approval was obtained from the Swedish Ethical Review Authority (ID 2020-06140). Amendments were approved: ID 2021-02434 and 2021-03755. All participants gave written informed consent after receiving both oral and written information. Confidentiality was assured.

### Setting

The project was undertaken in seven orthopaedic units (located at university, regional, and local hospitals across Sweden) constituting the intervention group in the OPTION trial [21]. Recruitment period for this study was between 15/12/2020 and 15/06/2021.

### The details of the implementation intervention

The implementation intervention entailed assigning, training, and supporting multiprofessional teams, internal to the intervention sites, for them to facilitate the uptake of clinical practice guidelines:

1. All intervention units were instructed to identify and assign members to a local IF team. Each team was to comprise at least one first-line manager, one registered nurse, one assistant nurse, and an occupational therapist or/and physiotherapist.
2. The IF teams were offered a 12-month support programme aiming to help them both to acquire knowledge about UR, primarily linked to the evidence-based CPG, and to understand how to bridge the know-do gap regarding bladder monitoring and prevention and treatment of UR in the orthopaedic context. The IF teams were also offered a variety of tools and resources to facilitate knowledge implementation in general, addressing the CPG implementation in particular.

For further details see Supplementary S1 File.

### Procedures

**Fig 1:**
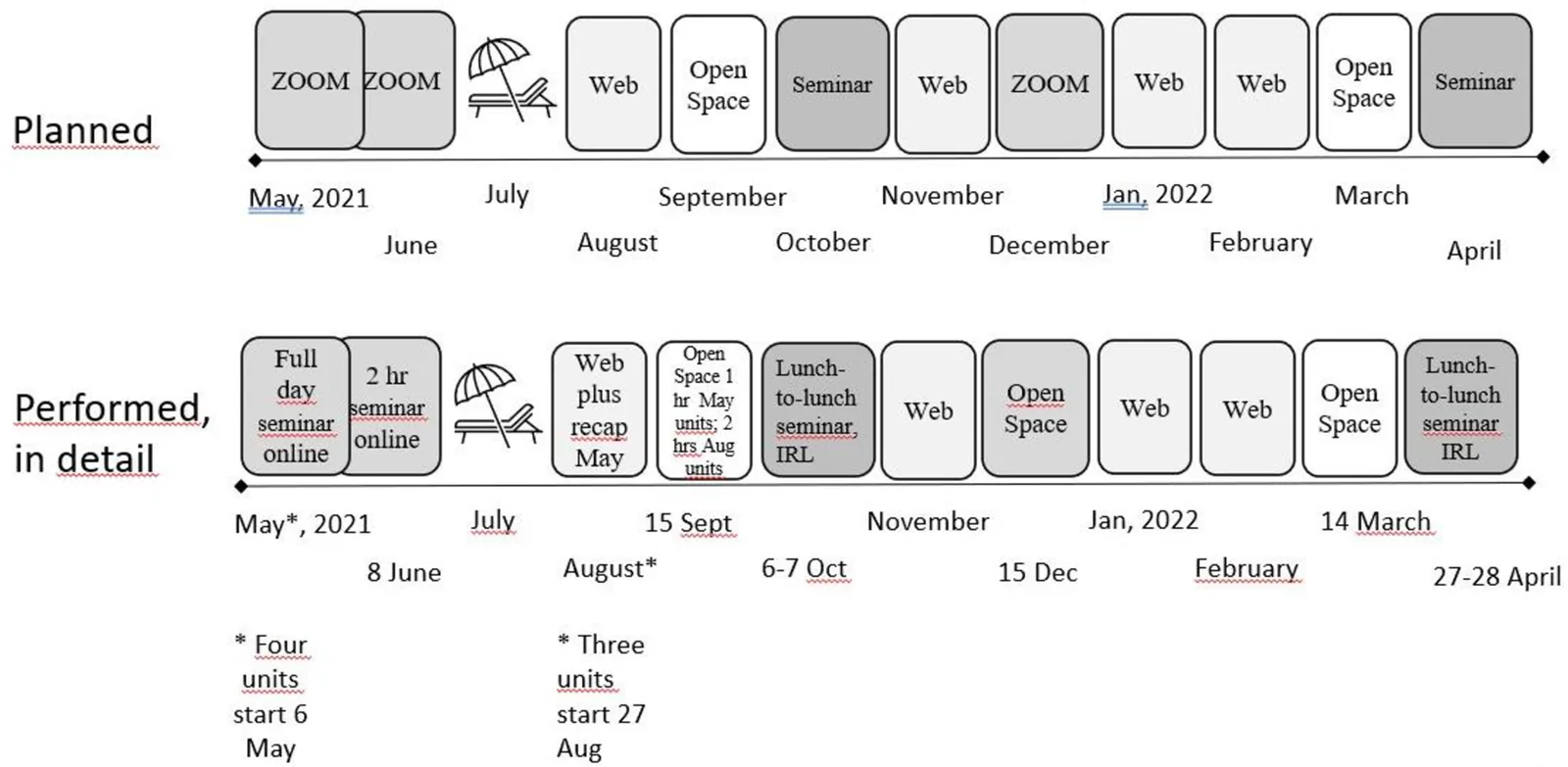
Overview of the planned and performed 12-month intervention.

The implementation intervention was delivered over a 12-month period and included face-to-face and digital seminars, digital meetings, digital open space support, and a digital mailbox for communication (Fig 1). It was delivered by three facilitators external to the organisations [31], experts in UR, knowledge implementation, and implementation leadership. All elements were linked, including the digital mailbox, which was surveyed for any e-mails/queries on these issues; IFs could raise questions and have a response within a week (including holidays).

The delivery of the implementation intervention was modified because of the COVID-19 pandemic travel restrictions. Specifically, the initial lunch-to-lunch seminar was converted into two digital seminars, and the start of the intervention was delayed by 3 months (to May 2021). Further, and again due to the pandemic, some intervention units were turned into COVID units during the first 6 months of 2021; these joined the intervention in August 2021 (and thus received one less opportunity to interact with the external experts in a virtual session called Open Space than those that joined the intervention in May 2021).

### Data collection

Data were collected from 06/05/2021 to 16/08/2023:

1. Semi-structured group interviews following the same guide with each of the seven IF teams when the implementation intervention was completed and again 1 year later. At that point, one team was no longer working but one former team member provided answers to the interview questions by e-mail. All other interviews were performed face-to-face or via video conference, led by two researchers not involved in the intervention. The interviews were digitally recorded and transcribed verbatim prior to analysis.
2. Facilitator activity logs were completed by 10 of the IFs, and photos were taken of the notes made by the IF teams on whiteboards during the intervention workshops.
3. Notes made by the external facilitators during the intervention and educational material from the implementation intervention were additional data sources.

### Data analysis

First, interview data were subjected to a theoretically driven thematic analysis in a realist approach [32]. Two authors, one separate from the intervention and one on the external facilitator team read all interview transcripts separately, familiarising themselves with the data, to get a sense of the whole. Then, two interviews from one unit were used to identify meaning units corresponding to the five initial hypotheses. These were contrasted and discussed with all researchers until consensus on the best interpretation. Subsequently, the remaining interview transcripts were analysed with the same approach. These meaning units were summarised for each initial hypothesis, representing what reflected context, mechanisms, and outcomes, respectively.

Once the interview data were completely exhausted, the IF activity logs were analysed with respect to further details on the context, mechanisms, or outcomes. Next, we incorporated the notes made by the external facilitators during the intervention, photos of notes made by the IF teams on whiteboards during the intervention workshops, and educational material procured by the external facilitators for the implementation intervention. These were also mapped with respect to context, mechanisms, and outcomes.

To conclude, the accrued findings were reconsidered with respect to the initial sense of the whole and emerging findings, anchoring yet developing a comprehensive understanding. By contrasting the comprehensive understanding with the initial hypotheses and the underpinning frameworks, we concluded five refined hypotheses.

Trustworthiness [33] was ensured by a parallel analysis of two authors and repeated critical reviews of the extended team.

## Results

The findings are represented by each of the initial hypotheses (as in Table 1), followed by a comprehensive understanding including the refined hypotheses.

### Findings corresponding to hypothesis 1

All IF teams had prior experience of practice and improvement training and execution. However, they experienced the OPTION programme (that is, the implementation intervention) as novel and unique as it combined both clinical and implementation knowledge. Initially, the IFs thought the support programme was complex, and the process described for knowledge implementation in general and CPG implementation in particular was less structured than they previously knew of. At this point, the prolonged and flexible structure of the programme raised concerns; the teams that stayed on went along with the process, gradually becoming more at ease with the programme. The IF teams were uncertain about what was expected of them and the purpose of the intervention. Two IF teams (28.6%) stopped participating in the programme following the first two components due to staffing issues. At follow-up, one of these teams expressed difficulties grasping the purpose of the programme while the other made no comment. The IF teams that completed the full programme (71.4%) developed more in-depth knowledge about bladder care, bladder monitoring, and the content of the national CPG. As a result, they were able to identify discrepancies between national and local guidelines. In addition, these IFs indicated that they had learned implementation strategies they had not known before, moving beyond their prior skills in quality improvement standard procedures. The IFs described increased confidence, enabling them to perform local activities such as planning and conducting training sessions and lectures, and preparing quizzes for their colleagues to engage them in changing bladder care practices.

### Findings corresponding to hypothesis 2

Not all IF teams comprised representation from each of the stipulated professions; some lacked either a first-line manager, a registered nurse, and/or an occupational therapist and/or a physiotherapist. While these teams collaborated with key stakeholders, they did not formally include them on their local IF teams. This was mainly due to the (common) matrix organisations, with the hip surgery trajectory including several professions, organised in different segments and with different managers, more or less engaged in the everyday healthcare processes. While the IF teams varied in size, from two to six members, some teams remained unchanged throughout the one-year intervention, while others added or lost team members. The smaller teams (that is, with two or three members) described being vulnerable to member loss.

Most teams (five out of seven, 71.4%) stayed with the entire implementation intervention programme. These IF teams indicated that the OPTION support programme made them realise the significance of letting the implementation process take time; understanding that there was no benefit in rushing the change (in favour of the adoption of the bladder guideline) eased their local implementation process. Allowing time made it possible to reach everyone on the staff, across all shifts. The IF teams that participated in the full one-year programme described their local teamwork and a sense of a shared mission, along with confidence in what they did was enabling the CPG implementation. Such team members identified themselves as role models in adhering to the CPG, and having a positive influence on their colleagues. They recognised that the adoption of the guideline could take some time, since implementation was recognised as a slow but ongoing process.

Being a team of IFs from different professions working together was beneficial in terms of members’ efforts to implement the CPG. In particular, this occurred in teams that included rehabilitation staff, enabling communication with regards to both rehabilitation and nursing issues. One team added a staff member from the postanaesthetic care unit, proposing this to enhance the likelihood of the guideline being adopted throughout the entire patient hip surgery trajectory. Some IFs described having to work through local resistance to change, although no team described a massive rejection of their local efforts. They experienced a lack of progress in the implementation of the CPG, due to lack of engagement of all units involved in the hip surgery trajectory.

### Findings corresponding to hypothesis 3

The teams varied in size and professional contribution; all members collaborated on equal terms, including manager(s) on the team. One team did not have a first-line manager as a member but had their verbal support for their local efforts. Further IF teams experienced resistance from other professions or units at their hospitals, who were not represented on the team. There were differences between the teams’ orthopaedic context, with some teams describing a general receptivity to change, others not. Further, some teams had to restart in planning of and performance of actions for the guideline implementation, as the local care shifted in relation to the pandemic phases. This—in addition to manager change and staff turnover within the IF teams—affected their processes.

First-line managers’ involvement strengthened the local teams; their managerial status gave authority to the team’s decisions, while the managers backed their IF team during the programme and beyond. The IF teams spent more time planning the guideline implementation—rather than their usual darting into action with a subsequent follow-up—as a response to the support programme. When faced with resistance to change, the IFs employed strategies learned through the programme, such as being resilient. To accomplish their implementation plans, the IFs also used change process tools that they were familiar with from previous training and quality improvement initiatives.

The teams easily adapted the CPG, with UR and bladder monitoring being described as relevant topics across all local segments. Still, reaching consensus on unit routines was challenging, but teams from smaller units described more efficient and effective routes to a joint decision. Furthermore, other staff in the units were considered likely to accept to the necessary changes for the CPG implementation, as they were proposed by their peers on the IF teams. Both the teams and their colleagues agreed that the guideline was relevant to the nursing and rehabilitation care of hip surgery patients.

### Findings corresponding to hypothesis 4

The IF teams found external facilitators easily available for e-mail and phone support to answer their questions. Additionally, the teams appreciated coming together with the other IF teams in the support programme, especially in the face-to-face events. The teams considered the external facilitator support essential to promoting their guideline implementation, helping them to reflect on local issues with CPG implementation from different perspectives. Being listened to and having the opportunity to discuss was significant to the teams.

The teams also identified further needs to progress implementation, and some teams made contact with each other between the programme events. Upon request from the teams, the external facilitators produced educational videos on the CPG, which were used for local in-house training at some of the sites, enabling the teams to proceed with implementation. Meeting with other IF teams was appreciated but did not facilitate their own, local implementation process – even if some tips and tricks were shared. No major collaboration was initiated and the contact ebbed away.

### Findings corresponding to hypothesis 5

The first-line managers on the local IF teams described their role in implementation as having the authority and power to make decisions, providing the staff with relevant conditions and time to work with implementation. Managers recognised their managerial support as crucial for implementation efforts, being particularly important at the beginning of an implementation process, whilst reinforcing the local teams’ assignment. Showing trust in their local IF team members and their abilities to implement the guideline, the first-line managers could allot time for their local implementation tasks, enabling team building and related work.

First-line mangers appreciated the opportunity to have dedicated time with the other IF team managers to learn about and discuss implementation leadership as a programme strategy. Even so, they expressed being of two minds as they also wanted to be with their IF team, working on their local implementation plan. First-line managers were more active at the beginning of the implementation process, until the IFs got hold of the project. None of the managers described altering their leadership activities or style as a result of the implementation intervention. The leadership role of an IF team varied, and was not necessarily tied to a formal management position, but any leader motivated the team and helped progress the implementation.

### A fostered comprehensive understanding of the hypotheses

The IF teams that stayed with the full OPTION support programme (71.4%) learned that not rushing implementation was a way to progress; the planning phase benefitted from a more detailed mapping of their local context, considering both barriers and enablers. The OPTION support programme provided them with a safe haven, and a shared understanding of the orthopaedic context across the IF teams. Further, it provided the IFs with what they considered a toolbox of implementation strategies, methods, and means, with introductions and further guidance from the external facilitators. While the toolbox initially differed much from the IF teams’ point of origin, that is, a more structured idea of quality improvement, this hampered their immediate recognition of the intervention programme.

The implementation of guidelines went beyond what the local teams expected at first; they learned that what they were doing was more than simply adopting new guidelines and that the process of making them part of the everyday routine required more work and more time. The IF teams still focused on planning and performing activities, both within the IF teams and for their peers, which gave them a sense of being in control, and of having accomplished something. They spent more time on the implementation, less on reflections on what worked and what did not work to progress the adoption of the CPG. Over time, the mapping of local barriers and enablers was revisited, providing for learning and a more tailored approach to implementation. By recognising such factors, the teams suggested a more sustainable guideline implementation.

The refined hypothesis I was framed as:

**Committed IFs (C), realising implementation components for their local context to adopt the bladder CPG (M), employ primary steps for improvement considering the local starting point through a one-year programme (O).**

The support programme’s educational sessions included and combined clinical and implementation knowledge. It informed the local teams about the content of the CPG, including lectures and clinical cases describing the consequences of bladder distention, appealing to norms such as the need for evidence-based practice. As the IFs moved beyond a general understanding of quality improvement, they gained a broader understanding of the implementation of knowledge into practice. The IF teams identified that they themselves had needed the further training, and that their colleagues had similar learning needs. Being provided with a tailored programme increased their trust in necessity of both learning about the CPG and having implementation support.

The IF teams recognised the novelty of the support programme: it combined knowledge about a particular CPG with knowledge about implementation. This combination helped the IFs develop the knowledge they gained was considered useful to promote improvements in many areas, even if the training on the bladder care CPG was specific to this project.

The refined hypothesis II was framed as:

**Known, contemporary, national clinical practice guidelines presented by the external facilitator in a pedagogic way (C), appealing to their everyday professional task and recognised for their importance for good and safe practice, with additional knowledge of implementation processes and conditions (M), enables internal facilitators’ understanding of the CPG as well as the complexity and variety of challenges of, and potential strategies for, guideline implementation (O).**

The IF team members’ background ensured an understanding of their particular local context, including its barriers to and enablers of the adoption of the guideline. This process was at times stressed by the changing context, with respect to the pandemic that altered the units’ staffing and patient situation. Moreover, the inclusion of managers was considered favourable to the IF teams; the managers represented trust and authority, establishing confidence in the IF team and reinforcing their peers’ ability to change practices in favour of the CPG. The joint programme enhanced their potential for collaboration on the necessary adoption of bladder care attention and procedures.

The refined hypothesis III was:

**Multiprofessional IF teams including a first-line manager or informal team leader (C) mandating knowledge implementation (M) seek to modify local context barriers (O).**

The IF teams described being more used to particular quality improvement processes. They preferred that the external facilitators suggested the agenda for each occasion in the support programme. Having support from the external facilitators, sharing their expertise and experience, helped the IF teams to keep momentum going during the during the one-year intervention. The external facilitators were considered trustworthy and collaborative, making themselves available for the IF teams whenever the teams reached out. The IF teams mostly used the face-to-face and digital meetings (over the mailbox and Open Space opportunities) for communication with the external facilitators, and mainly for CPG guidance.

A revised hypothesis IV reads:

**IF teams receiving regular support and guidance from external facilitators (C) understanding CPG implementation (M) address local barriers and enablers in planning and performing guideline implementation (O).**

All managers had considerable clinical and managerial experience. They were dedicated to their role as leaders and their assignment, recognising the importance of role-modelling in times of change. With joint training sessions for the managers on implementation leadership, there were time for reflection and opportunities to share experiences with fellow managers about leading change.

Describing a constant flow of change processes (dispatched from their upper hospital/regional organisations), their common procedures included reinforcing that efforts presumably aimed at quality improvement. The managers recognised the importance of supporting their staff, and during the programme, the managers targeted support of their IF team. The managers primarily emphasised relations- and task-oriented leadership, in that order, and collaborated with their local teams (rather than other managers) to facilitate the CPG implementation.

A refined hypothesis V reads:

**First-line managers provided with support on facilitative leadership (C) split between their team and their own responsibility and role (M) will understand but not necessarily act on their own attitude and conduct in knowledge implementation (O).**

## Discussion

This process evaluation was designed to investigate what worked, for whom, and why (or why not) in terms of facilitating implementation of an evidence-based CPG for bladder care in orthopaedic nursing and rehabilitation. Though the OPTION intervention positively affected the attitudes of the IFs as individuals and teams, a subsequent adoption of the CPG was initiated in some of the intervention sites only. The evaluation provides a further understanding of staffs’ and managers’ experiences of the intervention, reflected below. Features discussed are: learning about and having support for guideline implementation; leadership components of knowledge implementation; and theoretical perspectives of implementation and leadership.

The knowledge-intensive sector of healthcare continually struggles to keep up with and promote evidence-based practices, which is further complicated by the complexity of such processes [34]. As such, healthcare needs to be, and to recognise itself as, a constantly learning organisation [35]. A learning organisation is characterised by investment in knowledge and development, at all levels, for the benefit of both innovation and creativity [36]. De Kok et al. propose five attributes for learning and improvement capabilities: perceived leadership commitment, open culture, room for team development, initiating and monitoring change, and strategic client focus [37]. Consequently, a learning organisation is expected to embrace guidelines as offering opportunities to improve and to identify the resources it needs for implementing evidence as the new way of doing things better [38]. Our findings reflect that the IF teams used collaborative attitudes and encouraging behaviours to create open cultures with room for reflection over an extended period of time, facilitated by the external facilitator support and guidance. Recognising the local teams’ shared experiences of the orthopaedic healthcare context (establishing the teams’ social capital), the intervention capitalised on the effect that linking between high social capital and implementation of knowledge can have [39]. Even so, working in distinct processes organised across separate sections of their larger healthcare organisations, the teams’ efforts were hampered by hierarchical leadership structures and a lack of alignment between strategic and operational priorities as well as an excessive workload and high staff turnover [37].

Prior training initiatives on quality improvement have been widely spread within the healthcare context, including the Swedish healthcare sector [40]. The IFs in our study described being familiar with and having experiences of quality improvement; this included the key principle of using one specific methodology that they were familiar with – the PDSA [41]. The approach constituted by implementation science was considered novel, and it took the IF teams and their managers some time to grasp how the theories and frameworks could be used to unravel potential mechanisms of change, explaining how and why a change may or may not occur [42]. During the one-year support programme, we found that the IFs went from feeling uncertain to gaining deeper knowledge and developing an understanding that implementation adds to, and moves beyond, quality improvement. This required time and benefitted only those teams that stayed engaged (71.4%). Similar to other studies that used facilitators for improved nursing and allied health practices [43,44], providing training and support for local staff to enable change is a challenging process. The OPTION IF teams emphasised the importance of using the team’s manager in situations where their managerial authority was deemed necessary to move the implementation process forward, supporting the notion that managers have a key position as mediators to evidence-based practice and knowledge implementation [45]. Even so, the managers involved in the teams stepped back at times during the process, where other team members’ competencies were more valuable considering local contextual factors. This strategy is in line with previous evidence of using clinical champions to address certain barriers and behaviours at the provider level [46]. As suggested by Årestedt et al., the external facilitators may be highly appreciated, yet internal facilitator teams can learn from each other too, encouraging a number of shared sessions [47]. While the role of external facilitator to some extent includes being a project manager, often with expertise on change processes and the ability to engage stakeholders [31], the proactive approach and offering contextually adapted support was beneficial to furthering the local implementation processes [48].

The i-PARIHS framework [23] provided the general frame for the intervention, rendering the forming of local teams to facilitate guideline implementation and learning of knowledge implementation for the benefit of this enterprise and further improvement initiatives. We found that the i-PARIHS aided in framing the hypotheses, and in designing the intervention components and structure. Still, prior pilot studies in the Swedish health and residential care context have indicated that addressing only managers with no additional internal facilitators [49,50], or vice versa [18], hampered CPG implementation. Thus, by attaching leadership components to the OPTION intervention, we primarily suggested an expansion of the leadership role to facilitate facilitators. Both i-PARIHS [23] and the O-MILe[24] recognise successful implementation as a quest for evidence matching the context, and further, they recognise that strategies addressing both the evidence and context are required. To shape and meet the characteristics of guideline implementation, the O-MILe framework also articulates a translation process, yet it points out the need to engage managers, and the need for those managers to balance task-, relations-, and change-oriented behaviours in alignment with the context needs and implementation process calls. Earlier research suggests that first-line managers intuitively use behaviours in accordance with O-MILe, yet mainly acting on their own experiences of what has worked in the past [51]. Further, they can appreciate theoretical support for their implementation leadership actions, strengthening behaviours in favour of implementation [52]. We found that supplementing i-PARIHS with O-MILe for the leadership components enabled a further exploration of factors important for the intervention support programme. However, neither the IFs nor the managers at the intervention sites were familiar with discussing implementation management and processes using these (or other) theories, models, or frameworks. We suggest that a transparent link with clinically sustained and well-established frameworks such as the i-PARIHS and O-MILe enabled further learning and discussion opportunities, both within the intervention and during the analysis and reporting phases of the OPTION process evaluation [53].

### Limitations

While the study and the OPTION support programme took place during the COVID-19 pandemic, minor adaptations in line with pandemic restrictions and impact were necessary. Firstly, full adherence to the randomisation was not possible, as units were altered in terms of patient care and staff assignments. Secondly, the data collection had to be adjusted: rather than observations of the uptake of the intervention, stalled by the visitor restrictions, we had to settle for interviews. Non-participant observations are suggested as a natural route to access everyday events and interactions [54], with opportunities to identify disparities between reported practice (which can be inflicted by social desirability) and healthcare procedures [55]. Consequently, we conducted a process evaluation rather than a full realist evaluation, relying chiefly on the team interviews and activity logs. This may have limited the full representation of mechanisms and outcomes. Further, the leadership components may or may not be less addressed as a result of the first-line managers being interviewed together with their employees on the IF teams. Several managers played down their leadership role in general and emphasised their collaboration with fellow team members as being of primary importance. Prior studies have suggested that joint efforts are needed, but further research is required to identify the optimal balance between leadership support and progress of the individuals and the teams [56]. While blinding the interviewers from both the randomisation outcome, and the implementation intervention structure and content, there were limited opportunities for them to expand on intervention experiences. However, this supplemented a focus on the teams’ experiences and trajectories, with limited risk of biased reports [57].

The training and support programme relied on the availability of external facilitators, which impacts the potential for scaling up [58]. The external facilitators also engaged in the analyses and refinement of hypotheses, requiring repeated discussions within and beyond the research team. In this case, the external advisory board served as critical friends to support reflexivity and prevent interpretation bias. Also, our study lacked opportunities to establish the cost-effectiveness of the IF team interactions with one another, other teams, and the external facilitators, as no data was collated on the time and efforts spent between the organised training and support program events. All in all, these limitations call for a careful translation of the findings to different conditions and contexts, particularly with respect to the data collection limitations caused by the pandemic. Rather a further effort to tailor a programme for the generic factors of the orthopaedic context is needed, taking advantage of the OPTION study outcomes.

## Conclusions

This process evaluation found primary steps of guideline implementation initiated by the local IF teams learning of principles for knowledge implementation, and their further comprehension of the guideline’s content and purpose. The findings are offered for future clinical trials and theoretical development, particularly indicating that a team effort including staff and managers’ sustained roles, resources, and mandate are required. We found the external, tailored support beneficial, though indicative of further needs to scale up an efficient way to reach out to multiple orthopaedic sites. A comprehensive adoption of the bladder care guidelines across the orthopaedic care context requires further or prolonged efforts; this is a slow process, calling for extended follow-up to catch long-term implementation intervention outcomes and effective use of resources.

## Supporting information

S1 File Supplementary table 1. Overview of the content of the OPTION implementation intervention.
(DOCX)

S2 File Rameses II checklist. Rameses II reporting standards for realist evaluations.
(DOCX)

S3 File StaRI-checklist-for-author-completion. Standards for Reporting Implementations Studies (StaRI).
(DOCX)

S4 File PLOSOne_Human_Subjects_Research_Checklist. (DOCX)

## Abbreviations

C: Context
CMO: Context-Mechanism-Outcome
CPG: clinical practice guideline
IF: internal facilitators
i-PARIHS: integrated Promoting Action on Research Implementation in Health Services
M: Mechanism
O: Outcome
O-MILe: Ottawa Model of Implementation Leadership
OPTION: Onset PrevenTIon of urinary retention in Orthopaedic Nursing and rehabilitation
PDSA: plan-do-study-act
RAMESES II: Realist And Meta-narrative Evidence Syntheses: Evolving Studies II
RE: realist evaluation
StaRI: Standards for Reporting Implementation Studies
UR: urinary retention.
