# Supplementary material for: Implementation of evidence-based practice guidelines to prevent urinary retention in hospitals: A process evaluation in orthopaedic care
Source: PLoS One. 2026 Sep 23;21(9):e0338755. doi: 10.1371/journal.pone.0338755 (PMC13600508; doi:10.1371/journal.pone.0338755)
Supplement: Supplementary file 1 [file pone.0338755.s001.docx]

| **Part of intervention** | **Specific aim** | **Participants** | **Evidence applied** | **Time frame** | **Date** |
| --- | --- | --- | --- | --- | --- |
| Seminar 1 | - To portray what is known to facilitate successful implementation of evidence in practice - To share and discuss the potential barriers and facilitators of implementing urine retention (UR) evidence in orthopaedic nursing and rehabilitation. - To provide knowledge of anatomy and physiology of the urine system, risks for UR associated with hip surgery - Provide the evidence on how to assess and manage UR and/or risk for UR in acute care. - Sharing leadership attitudes, actions and behaviour to facilitate guideline implementation | Internal facilitators (IFs) and their first line managers | The integrated Promoting Action on Research Implementation in Health Services (i-PARIHS) framework. The Plan-Do-Study-Act, and the Knowledge-to-Action (KTA).  National and international evidence on UR, the Ottawa Model of Implementation Leadership (O-MILe). Local data from baseline illustrating current UR practice in the intervention units. | One full day (digital) | 6 May 2021 |
| IF assignment | - Start mapping local context, constructing a plan for overcoming barriers and taking advantage of facilitating aspects and experiences | IFs sponsored by their first line managers | The i-PARIHS framework.  The KTA-Cycle.  National and international evidence on UR (as above) | 8-16 hrs of allocated time + e-meetings with IFs and external facilitators |  |
| Seminar 2 | - To provide a deeper understanding for the complexity of urinating and urinary complications - To provide a deeper understanding of context mapping and planning for implementation | IFs and their first line managers | As above | 2 hrs Web-seminar | 8 June 2021 |
| IF assignment | - Further mapping local context, constructing a plan for overcoming barriers and taking advantage of facilitating aspects and experiences | IFs sponsored by their first line managers | As above | 8-16 hrs of allocated time + e-meetings with IFs and external facilitators |  |
| Seminar 3 | - Discuss local plans and further explore strategies | IFs and their first line managers | As above | Lunch-to-lunch seminar IRL | 6-7 October 2021 |
| IF assignment | - Completion and performing local plans for implementing UR guidelines | IFs sponsored by their first line managers | As above | 8-16 hrs of allocated time + e-meetings with IFs and external facilitators |  |
| Prolonged support | - To support the IFs in the ongoing implementation process | IFs sponsored by their first line managers | The i-PARIHS framework.  The PDSA and KTA-Cycle.  National and international evidence on UR | Monthly Web-meetings or OpenSpace with IFs and external facilitators | Nov 2021-March 2022 |
| Seminar 4 | - Experiences of implementing UR guidelines, sustaining change and continuous knowledge translation | IFs and their first line managers | As above + O-MILe | Lunch-to-lunch seminar IRL | 27-27 April 2022 |
